# Immediate inflammatory response to mechanical circulatory support in a porcine model of severe cardiogenic shock

**DOI:** 10.1186/s40635-024-00625-8

**Published:** 2024-04-22

**Authors:** Emilie Gregers, Peter H. Frederiksen, Nanna L. J. Udesen, Louise Linde, Ann Banke, Amalie L. Povlsen, Jeppe P. Larsen, Christian Hassager, Lisette O. Jensen, Jens F. Lassen, Henrik Schmidt, Hanne B. Ravn, Peter M. H. Heegaard, Jacob E. Møller

**Affiliations:** 1 Department of Cardiology, The Heart Center, Copenhagen University Hospital Rigshospitalet, Blegdamsvej 9, 2100 Copenhagen O, Denmark; 2 Department of Cardiology, Odense University Hospital, Odense, Denmark; 3 Department of Cardiothoracic Anesthesiology, Odense University Hospital, Odense, Denmark; 4 Department of Health Technology, Technical University of Denmark, Lyngby, Denmark

**Keywords:** Venoarterial extracorporeal membrane oxygenation, Cardiogenic shock, Acute myocardial infarction, ECMO, ECMELLA, Unloading of the left ventricle

## Abstract

**Background:**

In selected cases of cardiogenic shock, veno-arterial extracorporeal membrane oxygenation (V-A ECMO) is combined with trans valvular micro axial flow pumps (ECMELLA). Observational studies indicate that ECMELLA may reduce mortality but exposing the patient to two advanced mechanical support devices may affect the early inflammatory response. We aimed to explore inflammatory biomarkers in a porcine cardiogenic shock model managed with V-A ECMO or ECMELLA.

**Methods:**

Fourteen landrace pigs had acute myocardial infarction-induced cardiogenic shock with minimal arterial pulsatility by microsphere embolization and were afterwards managed 1:1 with either V-A ECMO or ECMELLA for 4 h. Serial blood samples were drawn hourly and analyzed for serum concentrations of interleukin 6 (IL-6), IL-8, tumor necrosis factor alpha, and serum amyloid A (SAA).

**Results:**

An increase in IL-6, IL-8, and SAA levels was observed during the experiment for both groups. At 2–4 h of support, IL-6 levels were higher in ECMELLA compared to V-A ECMO animals (difference: 1416 pg/ml, 1278 pg/ml, and 1030 pg/ml). SAA levels were higher in ECMELLA animals after 3 and 4 h of support (difference: 401 ng/ml and 524 ng/ml) and a significant treatment-by-time effect of ECMELLA on SAA was identified (*p* = 0.04). No statistical significant between-group differences were observed in carotid artery blood flow, urine output, and lactate levels.

**Conclusions:**

Left ventricular unloading with Impella during V-A ECMO resulted in a more extensive inflammatory reaction despite similar end-organ perfusion.

**Supplementary Information:**

The online version contains supplementary material available at 10.1186/s40635-024-00625-8.

## Background

Mortality after cardiogenic shock (CS) has decreased over time but is still associated with a mortality of 34–56% in patients with CS due to acute myocardial infarction [1, 2]. Mechanical circulatory support (MCS) is increasingly used as a bridge to recovery in CS refractory to medical treatment [3]. Different types of MCS are available, including intra-aortic balloon pump (IABP), veno-arterial extracorporeal membrane oxygenation (V-A ECMO), and micro-axial flow pumps (mAFP) [3]. Since the neutral IABP-shock 2 study was published, the use of IABP has decreased [4, 5]. Two small studies on early V-A ECMO treatment compared with standard therapy have recently been published. Both studies were neutral on the primary end-point, and recently, the adequately powered ECLS–SHOCK trial also failed to demonstrate benefit of routine V-A ECMO use in acute myocardial infarction CS [6–8]. Despite this, a considerable increase in use of mAFP (such as the Impella device) and V-A ECMO has been observed [5, 9]. Not infrequently these devices are combined in cases with inadequate left ventricular (LV) emptying on V-A ECMO. Observational studies indicate that V-A ECMO in combination with Impella (ECMELLA) is associated with improved outcome, but the combination has been associated with increased rates of serious complications [10, 11]. Cardiogenic shock is by itself often associated with a strong inflammatory response. Initiation of V-A ECMO may trigger an immediate inflammatory reaction, which can further destabilize the CS patient by promoting the inflammation already present due to tissue injury caused by the end-organ hypoperfusion fundamental for CS [12, 13]. It may be speculated that the addition of an extra mechanical device during ECMELLA may further enhance this inflammatory reaction.

We hypothesize that left ventricle unloading with Impella during V-A ECMO in case of CS enhances an inflammatory reaction. To test this, serial measurements of inflammatory biomarkers were obtained in a porcine model of CS treated with either V-A ECMO alone or ECMELLA.

## Methods

**Fig. 1:**
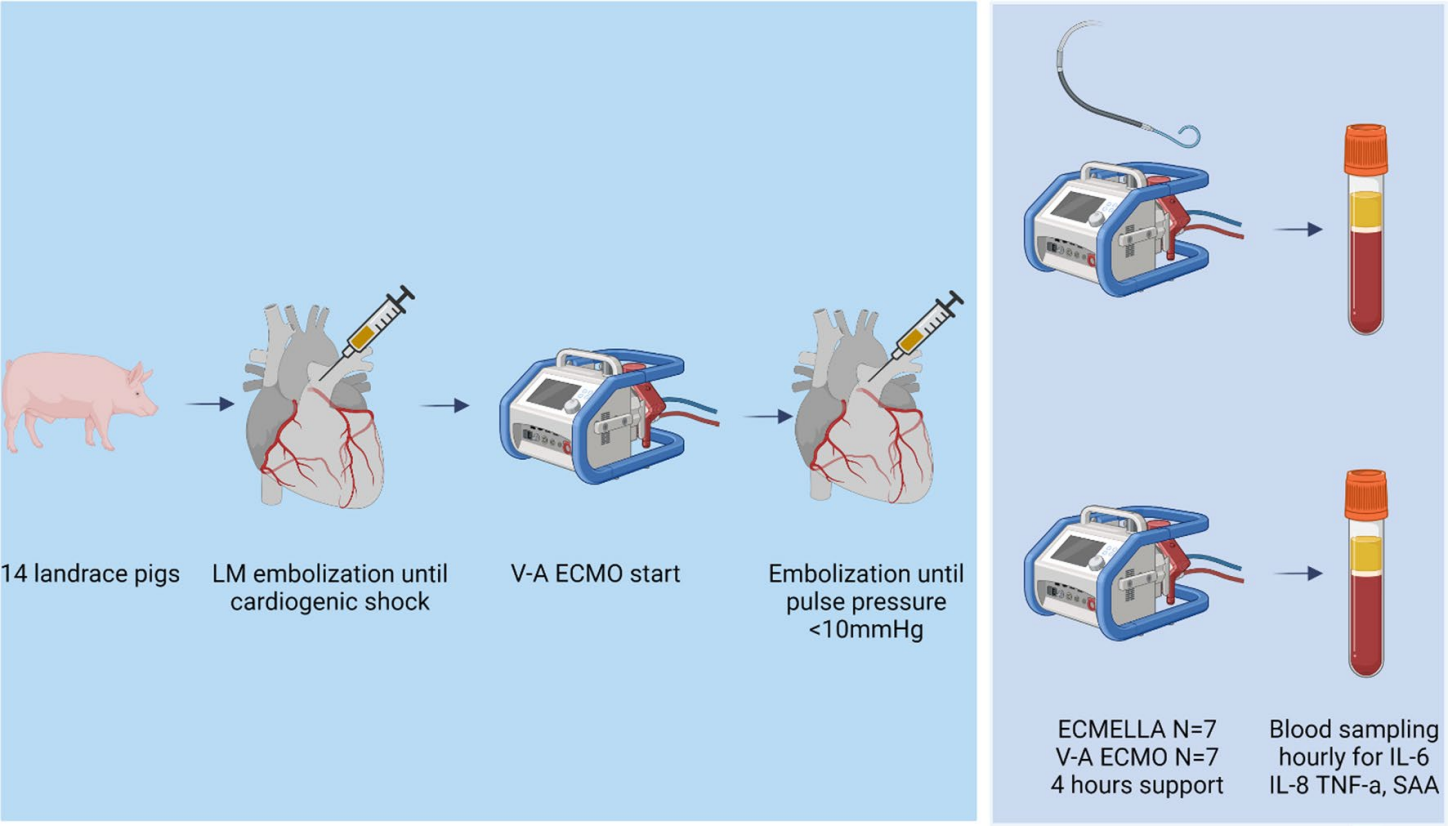
Overview of study setup. *LM* left main coronary artery, *V-A ECMO* veno-aterial extracorporeal membrane oxygenation, *ECMELLA* combined VA-ECMO and Impella, *IL-6* interleukin-6, *IL-8* interleukin 8, *TNF-α* tumor necrosis factor alpha, *SAA* serum amyloid A

We studied fourteen Danish female landrace pigs. The model has previously been described in detail by our group [14], and an overview of the study setup is available in Fig. 1. In short, all animals were anesthetized and mechanically ventilated. Amiodarone was used to limit malignant, irreversible arrhythmias. We have previously in the model observed a potent inflammatory reaction leading to uncontrolled vasodilation, capillary leakage, malignant arrhythmias, and early termination of the experiment during induction of shock and immediately after initiation of mechanical circulatory support. To circumvent this otherwise uncontrolled inflammatory reaction, 80 mg methylprednisolone was administered at study start in all but the first three animals.

### Instrumentation

All instrumentations were done percutaneously except for measurements of flow in the carotid artery where surgical cut-down was used to place a 4 mm Doppler probe (MEDSTIM SonoQ TTFM probe, Emtec GmbH, Finning, Germany) and the surgical preparation of a subxiphoid pouch for echocardiography. A pulmonary artery catheter was used for measurements of cardiac output (CO), mixed venous saturation (SvO_2_), and pulmonary artery pressure. Carotid artery cannulation was used for direct measurements of arterial pressure and for introduction of a conductance catheter in the left ventricle (Ventri-cath 510 PV Loop Catheter, Millar Inc., Texas, USA). A 17 French (Fr) cannula was placed in the left femoral artery and a 21 Fr cannula in the right femoral vein for V-A ECMO support. A sheath was placed in the right femoral artery for coronary angiography and embolizations in all animals and for Impella placement in ECMELLA animals. At last, a balloon-tipped catheter was placed in the left renal vein for blood sampling and pressure measurements.

### Intervention

Acute myocardial infarction was introduced by serial embolization of the left main coronary artery by injection of polyvinyl microspheres (Contour™, Boston Scientific, Marlborough, USA). Embolization was repeated until CS. We defined CS as a 50% reduction in CO, 50% reduction in SvO_2_, or an absolute SvO_2_ of < 30%. ECMELLA animals required 15 (12–17) embolizations and ECMO animals required 8 (5–10). Norepinephrine infusion was initiated if mean arterial blood pressure fell below 45 mmHg. After achieving CS, V-A ECMO was initiated at a flow of 50 ml/kg/min. Embolization was continued until the arterial pulse pressure was below 10 mmHg or until pulse pressure was unchanged after three repeated embolizations. In ECMELLA animals, Impella was initiated when target pulse pressure was reached and set to highest possible performance level without suction events. V-A ECMO or ECMELLA was continued for 4 h before the animal was euthanized. Experiments were conducted randomly in blocks of two animals alternating between ECMO and ECMELLA. Blinding was not possible.

### Blood analyses

Blood samples were drawn at baseline, at CS, and 1, 2, 3, and 4 h after V-A ECMO initiation. Arterial, renal, and mixed venous blood were analyzed immediately using epoc^®^ Blood Analysis System (Siemens Healthineers, Erlangen, Germany). Venous blood was separated into serum and frozen in liquid nitrogen before storage in a minus 80 ºC freezer until collective analyses could be performed.

We measured serum levels of interleukin 6 (IL-6), IL-8, Tumor necrosis factor alpha (TNF-α) and serum amyloid A (SAA). IL-6 and -8 concentrations were determined by ELISAs from the R&D systems (Duosets DY686 and DY535, respectively) which use goat anti-pig IL6/-8 for detection. Samples were run in duplicates in a 1:2 dilution with a detection limit of 62.5 pg/ml. TNF-α concentrations were determined by Invitrogen ELISA (Swine TNF-α cytoset CSC1753) which uses biotinylated anti-pig TNF-α for detection. Samples were run in duplicates in a 1:2 dilution with a detection limit of 125 pg/ml. Finally, SAA concentrations were determined using a commercially available sandwich ELISA (Phase SAA assay, Tridelta Development Ltd., Kildare, Ireland). Samples were tested according to manufacturer’s instructions except that the lowest dilution was 1:5 to increase signal intensity (detection limit of 78.15 ng/ml).

The study was approved by the Danish Animal Experiments Inspectorate (2006-15-00951) and conducted in accordance with their guidelines.

### Statistical analysis

The study was exploratory and no specific sample size estimation was performed for the current study. Continuous variables are presented as median with quartiles (Q1–Q3) with differences tested by Mann–Whitney *U* test. Categorical values are reported as n (%) and differences tested by Fisher exact test. Biomarkers of inflammation were analyzed by baseline (shock) corrected repeated measurement mixed model (R package “mmrm”) and reported as predicted means with confidence intervals and *p* values for both treatment-by-time interactions and group differences at corresponding time points. IL-8 and TNF-ɑ were log2-transformed and reported as back-transformed predicted means. Possible relationships between the inflammatory biomarkers and the degree of shock were investigated using Spearman’s correlation analyses of SvO_2_ and cardiac power output (CPO) at cardiogenic shock and peak inflammatory biomarker levels. CPO was calculated as $\frac{{\text{mean}}\;{\text{arterial}}\;{\text{blood}}\;{\text{pressure}}*{\text{cardiac}}\;{\text{output}}}{451}$. A *p* value < 0.05 was considered significant in all analyses. All statistical analyses were done in R version 3.6.1 (R Core Team, R Foundation for Statistical Computing, Vienna, Austria).

## Results

Fourteen consecutive landrace pigs weighing a median of 72 kg (Q1–Q3: 70–73 kg) with induced CS were managed with V-A ECMO (*N* = 7) or ECMELLA (*N* = 7). The animals were similar at baseline (Table 1). In five V-A ECMO animals and six ECMELLA animals, methylprednisolone was given prior to CS induction. All animals completed 4 h of mechanical support. At time of shock median CO (2.2 vs. 2.5 L/min), SvO_2_ (28% vs. 36%), and mean arterial pressure (MAP: 36 vs. 43 mmHg) were not significantly different between ECMELLA supported and V-A ECMO supported animals (Table 1). CPO was 0.9 W at baseline and decreased to 0.3 W in V-A ECMO and 0.1 W in ECMELLA animals when V-A ECMO was initiated. Five V-A ECMO animals developed ventricular fibrillation and were successfully cardioverted as opposed to none of the ECMELLA animals (*p* = 0.02). All but one episode occurred after initiation of MCS. We found no difference in the number of animals requiring norepinephrine (3 vs. 3). Median V-A ECMO flow after 1 h was 3.3 and 3.4 L/min. Estimated Impella flow after 1 h in the ECMELLA group was 1.0 L/min.

**Table 1:** Animal study characteristics

	Baseline		Cardiogenic shock		MCS 1 h		MCS 2 h		MCS 3 h		MCS 4 h	
	ECMELLA	ECMO	ECMELLA	ECMO	ECMELLA	ECMO	ECMELLA	ECMO	ECMELLA	ECMO	ECMELLA	ECMO
Cardiac output (L/min)	5.4 (5.2–5.6)	6.0 (5.6–6.3)	2.2 (1.8–2.4)	2.5 (2.3–3.3)								
Heart rate (beats per minute)	75 (66–82)	83 (78–84)	70 (66–72)	78 (75–80)	60 (58–64)	70 (67–86)	57 (53–58)	64 (59–68)	53 (47–58)	65 (63–80)	50 (43–58)	63 (61–72)
Mean arterial pressure (mmHg)	72 (64–88)	73 (62–79)	36 (33–44)	43 (36–48)	71 (66–77)	57 (45–62)	82 (72–88)	65 (64–74)	84 (72–91)	69 (57–82)	72 (60–83)	63 (56–72)
Pulse pressure (mmHg)	47 (32–53)	39 (32–40)	9 (8–10)	10 (10–14)	6 (6–6)	10 (6–12)	6 (4–10)	8 (8–8)	6 (6–10)	9 (8–12)	6 (5–6)	11 (9–12)
Cardiac power output (W)	0.9 (0.7–1.0)	1.0 (0.8–1.1)	0.1 (0.1–0.2)	0.3 (0.2–0.3)								
Mean pulmonary artery pressure (mmHg)	26 (26–30)	23 (20–26)	31 (26–32)	26 (23–28)	26 (18–30)	24 (18–29)	27 (17–28)	24 (21–29)	28 (20–30)	27 (24–33)	31 (23–33)	27 (22–32)
Pulmonary artery diastolic pressure (mmHg)	24 (21–27)	16 (15–19)	28 (22–30)	21 (20–22)	23 (23–28)	22 (18–26)	23 (16–23)	22 (19–25)	25 (23–28)	26 (20–30)	28 (21–28)	19 (18–30)
Left ventricle end-diastolic pressure (mmHg)	18 (17–19)	21 (20–22)	22 (21–22)	24 (20–26)	14 (11–15)	24 (20–27)	15 (13–18)	27 (24–29)	12 (11–14)	24 (23–28)	11 (10–15)	22 (18–28)
Mixed venous saturation (%)	62 (51–65)	65 (54–66)	28 (22–30)	36 (32–41)	67 (64–75)	60 (41–69)	71 (65–80)	59 (56–70)	75 (68–83)	58 (47–72)	72 (66–80)	75 (55–81)
Carotid artery flow (ml/min)	248 (226–261)	278 (249–301)	57 (24–86)	122 (98–144)	154 (116–271)	174 (162–211)	123 (116–268)	186 (174–222)	171 (141–248)	224 (176–248)	250 (134–316)	248 (208–297)
Renal venous pressure (mmHg)	6 (6–9)	10 (10–11)	7 (6–10)	13 (10–16)	6 (5–7)	8 (7–10)	6 (5–6)	11 (8–12)	7 (6–8)	10 (8–12)	7 (6–7)	11 (8–12)
Renal venous saturation (%)	82 (69–85)	75 (71–76)	39 (26–51)	30 (30–59)	87 (83–91)	77 (61–93)	87 (73–88)	57 (50–97)	86 (86–91)	84 (51–96)	74 (56–83)	71 (62–96)
Urine output (ml/h)	–	–	–	–	285 (185–475)	160 (85–270)	110 (57–330)	60 (49–72)	65 (40–272)	82 (62–102)	105 (42–273)	50 (16–144)
Norepinephrine (µg/kg/min)	0 (0–0)	0 (0–0)	0 (0–0.02)	0 (0–0)	0 (0–0)	0 (0–0)	0 (0–0)	0 (0–0)	0 (0–0)	0 (0–0.07)	0 (0–0)	0 (0–0.04)
V-A ECMO flow (L/min)	–	–	3.4 (3.4–3.4)	3.3 (3.2–3.4)	3.3 (3.1–3.4)	3.4 (3.4–3.7)	3.3 (3.0–3.5)	3.5 (3.4–3.8)	3.3 (3.1–3.6)	3.6 (3.4–4.0)	3.5 (3.0–3.7)	3.5 (3.4–4.1)
Impella flow (L/min)	–	–	–	–	1.0 (0.9–2.0)	–	1.8 (1.0–2.3)	–	1.9 (1.4–2.3)	–	1.7 (1.3–2.4)	–
Blood lacate (mmol/L)	1.3 (0.8–2.3)	0.9 (0.9–1.1)	2.5 (2.2–2.9)	2.1 (1.0–2.6)	3.4 (3.0–3.6)	2.7 (2.6–4.4)	3.7 (3.4–4.3)	3.5 (2.7–4.4)	3.4 (3.2–4.1)	4.6 (3.5–5.9)	3.5 (2.8–4.0)	4.4 (3.6–5.4)

### End-organ perfusion

At CS, cerebral perfusion reflected in carotid artery blood flow was more than halved from baseline with no significant between-group difference (248–57 ml/min vs. 278–122 ml/min). By 4 h of MCS, median carotid artery blood flow was restored to baseline level in both groups (250 vs 248 ml/min). Urine output declined equally in both groups during MCS with a gradual decline in hourly urine output over time. General tissue perfusion, reflected in blood lactate level, was affected during MCS in both groups. Lactate reached a plateau after 1 h of MCS in the ECMELLA group and after 3 h of MCS in the V-A ECMO group. Median peak lactate level was 3.4 mmol/l. There were no significant between-group differences in lactate levels at any time point. Left ventricle end-diastolic pressure (LVEDP) was consistently lower in the ECMELLA compared to the V-A ECMO group during MCS (Table 1).

### Inflammation

**Fig. 2:**
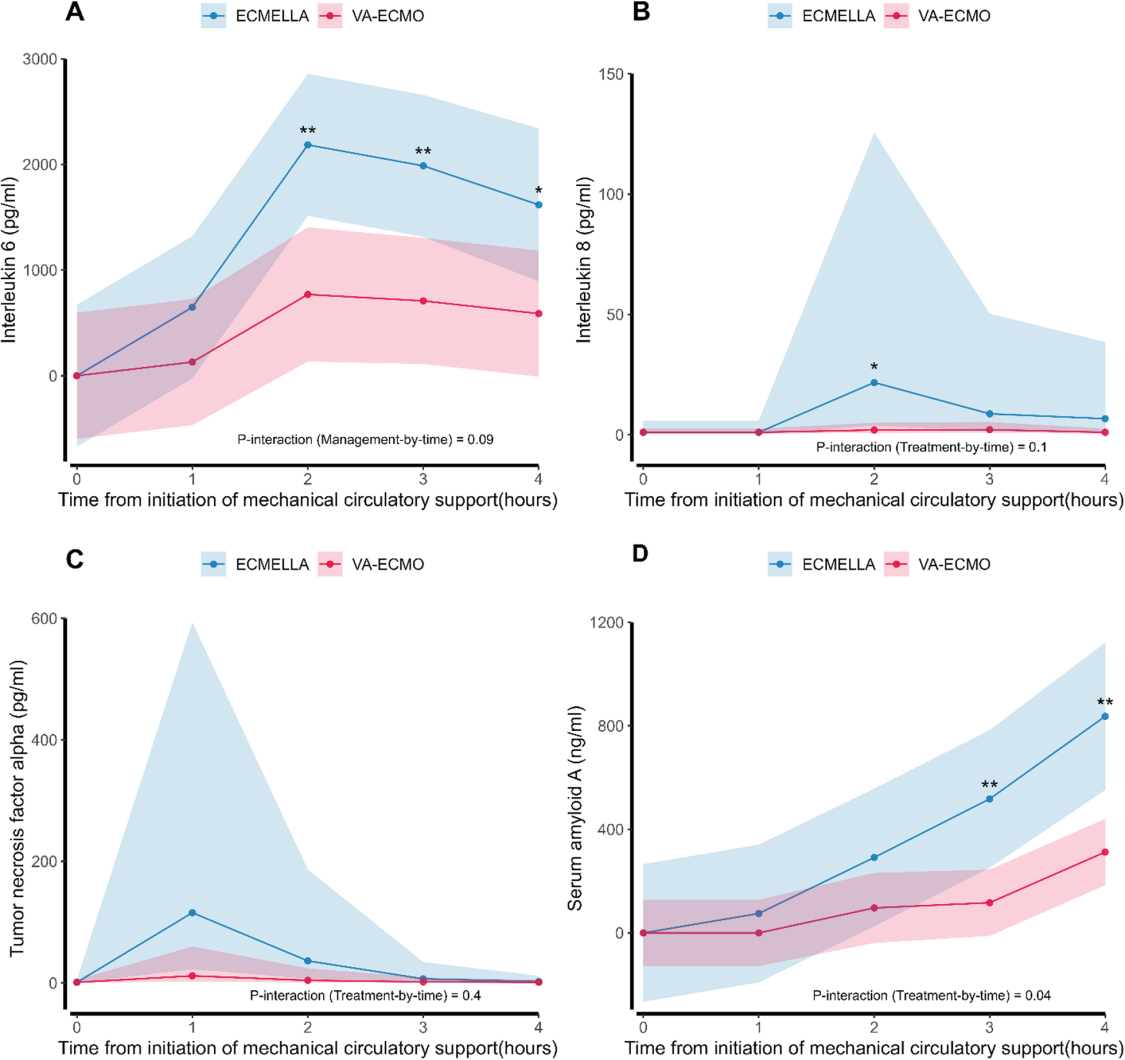
Fluctuations in biomarkers of inflammation after mechanical circulatory support (MCS) initiation. Mixed linear models comparing animals managed with V-A ECMO and ECMELLA in levels of inflammatory biomarkers over time from MCS initiation. **A** Interleukin-6 (IL-6), **B** Interleukin-8 (IL-8), **C** tumor Necrosis Factor alpha (TNF-α), and **D** serum amyloid A (SAA). V-An ECMO, veno-arterial extracorporeal membrane oxygenation; ECMELLA, V-A ECMO and Impella. *p < 0.05 and **p < 0.01 for management (V-A ECMO vs. ECMELLA) at corresponding time points

We found no significant differences between cardiogenic shock and pre-shock values for any of the measured biomarkers. Serum IL-6 peaked after 2 h of MCS and stayed elevated for the rest of the experiment. In contrast, IL-8 had a significant transient rise around 2 h of MCS, TNF-α had a non-significant transient rise at 1 h of MCS, while SAA demonstrated a late response with significant elevation at 4 h (Fig. 2 and Table 2). The degree of shock, measured as CPO and SvO_2_ at initiation of V-A ECMO, was not associated with peak levels of IL-6, IL-8, TNF-α, or SAA (Additional file 1: Figures S1 and S2). We found no significant difference in the levels of any of the measured inflammatory biomarkers between animals treated with or without methylprednisolone at study initiation (Additional file 1: Figure S3).

**Table 2:** Overall changes in inflammatory biomarkers over time from baseline to 4 h of mechanical circulatory support (MCS)

	Pre-shock *N* = 14	Shock *N* = 14	MCS 1 h *N* = 14	MCS 2 h *N* = 14	MCS 3 h *N* = 14	MCS 4 h *N* = 14
Interleukin-6 (pg/ml)	62 (62–509)	62 (62–272)	512 (71–1106)	2080 (1424–3280)	1692 (1031–2480)	1489 (499–2590)
Interleukin-8 (pg/ml)	62 (62–62)	62 (62–62)	62 (62–62)	73 (62–120)	62 (62–84)	62 (62–62)
Serum amyloid A (ng/ml)	156 (156–156)	156 (156–156)	156 (156–156)	190 (156–595)	372 (199–494)	710 (308–938)
Tumor necrosis factor α (pg/ml)	125 (125–125)	125 (125–125)	244 (125–364)	125 (125–410)	125 (125–131)	125 (125–125)

### Effect of intervention on inflammation

ECMELLA animals had significantly higher serum concentrations of IL-6 at 2–4 h of MCS compared to V-A ECMO animals (Fig. 2A). Likewise, serum IL-8 concentrations were transiently higher in ECMELLA animals at 2 h of MCS, while no between-group difference was identified for TNF-α at any time points (Fig. 2B, andC). Finally, ECMELLA animals had significantly higher levels of serum SAA at 3 and 4 h of MCS compared to V-A ECMO animals (Fig. 2D). A significant treatment-by-time effect was identified for SAA levels (F(4, 31) = 2.8, *p* = 0.04).

## Discussion

The present translational study suggests that an inflammatory response to severe myocardial injury and severe hemodynamic instability can be detected as early as within 1 h after injury. Although there was no difference in end-organ perfusion between V-A ECMO and ECMELLA animals, ECMELLA treatment was associated with a more pronounced inflammatory response with higher levels of serum IL-6 and SAA during MCS.

### End-organ perfusion

The model mimics a situation with extreme shock and almost absent intrinsic LV function as can be encountered after refractory cardiac arrest or massive acute myocardial infarction and in some cases with severe non-ischemic heart failure. In these situations, systemic perfusion is maintained by the MCS system often in terms of V-A ECMO with no or minimal emptying of the LV. If blood flow through the lungs is minimal, SvO_2_ becomes unreliable in assessment of systemic perfusion. Thus, a more organ-specific assessment of perfusion was targeted in this study. We found no difference in neither renal venous saturation, in Doppler-assessed carotid artery flow, nor in lactate levels between ECMELLA and V-A ECMO animals. This is in agreement with human observational data comparing V-A ECMO and ECMELLA in refractory cardiac arrest patients where similar post-MCS lactate levels have been reported [15]. We found no between-group difference in carotid artery blood flow, which agrees with previous studies reporting no difference in hypoxic brain damage in patients managed with V-A ECMO or ECMELLA during CS or cardiac arrest [11, 15]. Contrary to our result of similar urine output in V-A ECMO and ECMELLA subjects, Schrage et al. found a higher frequency of renal replacement therapy in CS patients managed with ECMELLA [11]. The difference in renal replacement therapy between V-A ECMO and ECMELLA was not confirmed in cardiac arrest patients [15]. Overall, our results support that ECMELLA therapy neither compromise end-organ perfusion compared to V-A ECMO in CS nor create problems with differential oxygenation in this model.

### Inflammation

During the 4-h study period, we observed an early inflammatory response in all animals, which is also a significant component in the pathophysiology of human acute myocardial infarction-induced CS [13]. SAA are apolipoproteins secreted during the acute phase of inflammation and regulated by the proinflammatory cytokines IL-1, IL-6, and TNF-α in pigs as well as in humans [16]. We found that ECMELLA-treated animals had increased serum concentrations of SAA likely associated with the elevation of IL-6 and TNF-α, which both rose earlier than SAA and peaked around hours 1–2. This result is clinically interesting considering that a study of patients with myocardial infarction not treated with MCS found SAA prognostic for LV dysfunction and mortality [17]. IL-6 is secreted by macrophages and is an important inflammatory mediator of the acute phase response, where it stimulates protein synthesis and production of neutrophils. Both IL-6 and IL-8 level has been associated with multiorgan failure and mortality [18, 19]. Although we did not find an overall ECMELLA treatment-by-time effect on IL-6 and IL-8 levels, we found elevated IL-6 levels in ECMELLA animals at specific time points. Recently, the CLIP score, a new risk stratification score in CS, has been presented as an early decision tool in CS. The score still lacks external validation but is based on four biomarkers and IL-6 gave the second highest contribution to the score [20]. Considering our findings of elevated IL-6 levels in ECMELLA vs. V-A ECMO animals at certain time points, the score may be less applicable on CS patients managed with ECMELLA.

A previous in vitro study indicates an inflammatory response to cardiac wall stress [26]. Considering this, unloading of the left ventricle during V-A ECMO in CS might be expected to limit the inflammatory reaction. However, despite decoupling aortic and LV pressure and a consistent decrease in LVEDP for ECMELLA animals indicating sufficient unloading, we saw that the added instrumentation increased the inflammatory response.

While the degree of inflammation in CS is associated with mortality, it is unknown whether the inflammatory reaction is simply a reflection of the tissue ischemia and injury caused by the CS, a result of the MCS system itself, or whether inflammation has an independent role in mortality [21]. Thus, it is unknown whether the amplified inflammation caused by the additional introduction of Impella during V-A ECMO in CS management is harmful and whether suppression of the inflammatory reaction is beneficial. However, previous translational studies have found elevated IL-6 involved in inflammation-induced anemia due to suppression of erythropoiesis [22, 23]. In addition, the IMICA trial tested the effect of the IL-6 receptor antagonist tocilizumab in cardiac arrest patients and found that tocilizumab reduced systemic inflammation and myocardial injury [24]. Moreover, the authors found that blockade of IL-6 receptors reduced vasopressor and inotropy requirements [25].

Observational studies with high risk of selection bias and uncontrolled confounding indicate that ECMELLA is associated with reduced mortality after CS compared to management with V-A ECMO alone; however, ECMELLA is simultaneously associated with an increased rate of complications [10, 11]. The benefit or harm of ECMELLA is currently being addressed (clinicaltrials.gov identifier NCT05577195). In the current study, we show a more extensive inflammatory reaction in ECMELLA management compared to V-A ECMO, which may explain some of the increased rate of complications observed in ECMELLA management. Therefore, considering the anti-inflammatory and myocardial preservatory effect of IL-6 receptor blockage previously shown in cardiac arrest patients, IL-6 receptor blockage could be a focus for future research in translational science and in CS patients managed with ECMELLA. Considering the current research available and results from this study, we believe ECMELLA should be reserved selected cardiogenic shock patientens with signs of LV overload on V-A ECMO, until results of ongoing randomized trials are available.

### Strengths and limitations

The study was conducted in healthy related female Landrace pigs of similar age and size from the same farm reducing the number of animals necessary to identify differences in treatment effect. The study period is limited to 4 h of MCS and whether inflammatory differences persist beyond this point is not known. Though the difference in cardiac output, MAP and change in carotid artery flow was not statistically significantly different between groups, ECMELLA animals may have experienced slightly more severe cardiogenic shock which may account for some of the observed difference in inflammatory biomarkers. The identified effects of MCS type may differ between species which necessitates confirmation of our findings in human CS cases preferably over a longer period (days). Methylprednisolone was not administered in all animals.

## Conclusion

In a short-term model of severe cardiogenic shock, we found that 4 h of left ventricular unloading with Impella during V-A ECMO resulted in a more extensive inflammatory reaction than 4 h of V-A ECMO alone despite similar end-organ. The implication of an amplified inflammatory reaction is unknown.

### Supplementary Information

**Additional file 1. **Supplementary Figures.

## Data Availability

The data sets used and/or analysed during the current study are available from the corresponding author on reasonable request.

## Abbreviations

CO: Cardiac output
CPO: Cardiac power output
CS: Cardiogenic shock
ECMELLA: Veno-arterial extracorporeal membrane oxygenation combined with Impella
IL: Interleukin
IABP: Intra-aortic balloon pump
mAFP: Micro axial flow pumps
MAP: Mean arterial pressure
MCS: Mechanical circulatory support
SAA: Serum amyloid A
SvO_2_: Mixed venous saturation
TNF-ɑ: Tumor necrosis factor alpha
V-A ECMO: Veno-arterial extracorporeal membrane oxygenation
