# Supplementary material for: Immediate inflammatory response to mechanical circulatory support in a porcine model of severe cardiogenic shock
Source: Intensive Care Med Exp. 2024 Apr 22;12:39. doi: 10.1186/s40635-024-00625-8 (PMC11035503; doi:10.1186/s40635-024-00625-8)

**Figure S1. Correlation between cardiac power output (CPO) and peak values of inflammatory biomarkers after induction of cardiogenic shock**. A: Interleukin-6 (IL-6), B: Interleukin-8 (IL-8), C: Tumor Necrosis Factor alpha (TNF-α), and D: Serum amyloid A (SAA). Correlations tested using Spearman’s rho statistics.


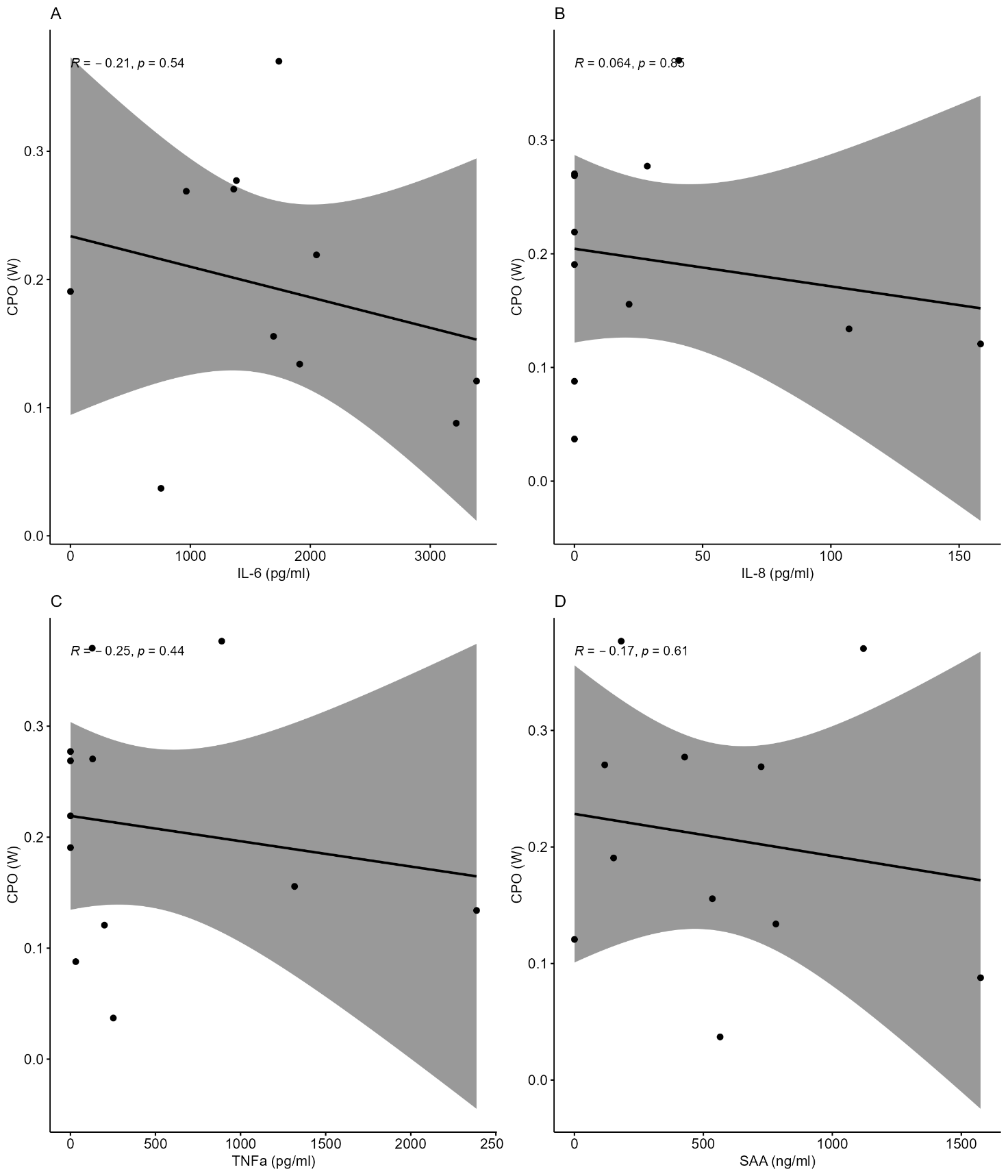


**Figure S2. Correlation between mixed venous saturation (SvO_2_) and peak values of inflammatory biomarkers after induction of cardiogenic shock**. A: Interleukin-6 (IL-6), B: Interleukin-8 (IL-8), C: Tumor Necrosis Factor alpha (TNF-α), and D: Serum amyloid A (SAA). Correlations tested using Spearman’s rho statistics.


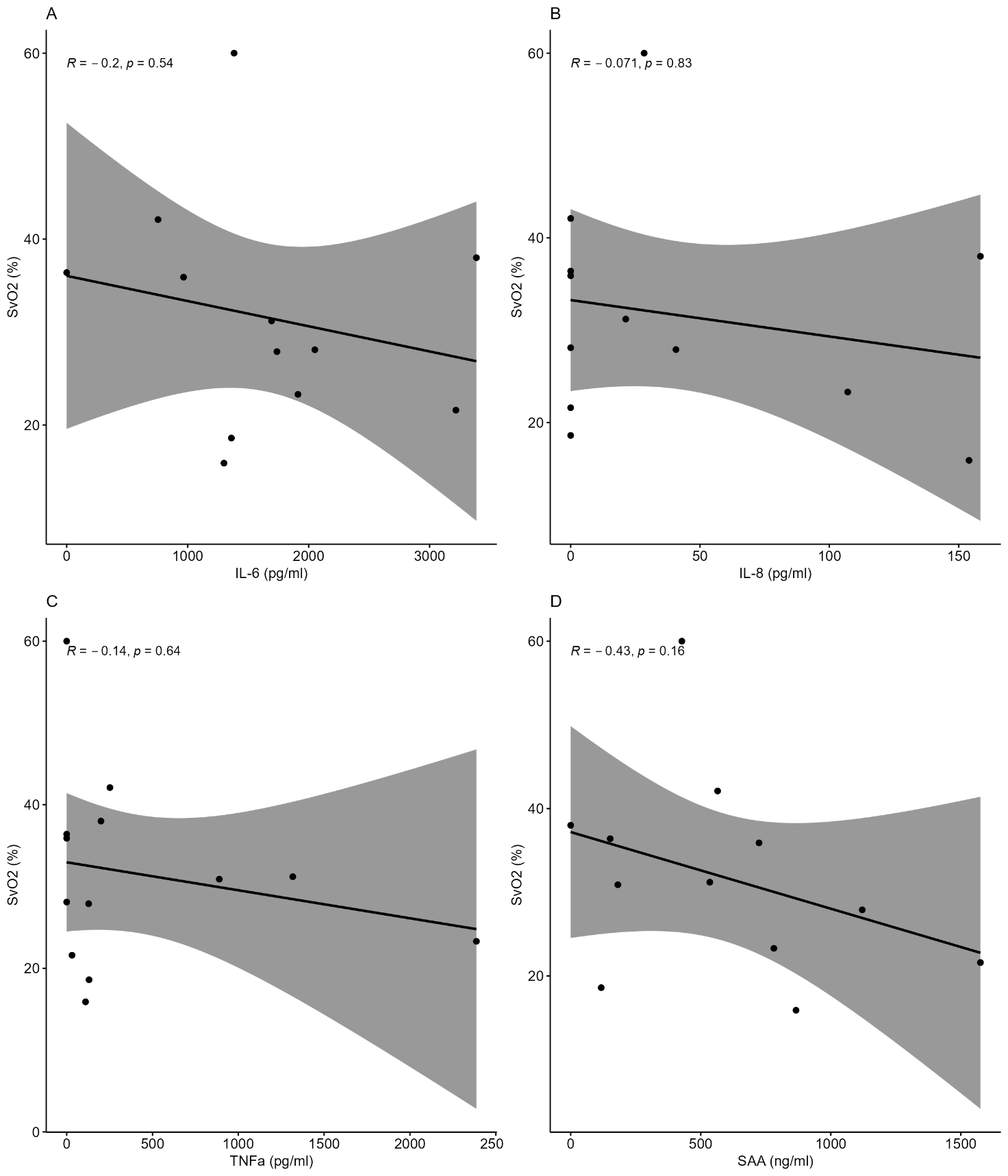


**Figure S3. Fluctuations in biomarkers of inflammation after mechanical circulatory support (MCS) initiation**. Mixed linear models comparing animals treated with or without methylprednisolone in levels of inflammatory biomarkers over time from MCS initiation. A: Interleukin-6 (IL-6), B: Interleukin-8 (IL-8), C: Tumor Necrosis Factor alpha (TNF-α), and D: Serum amyloid A (SAA).


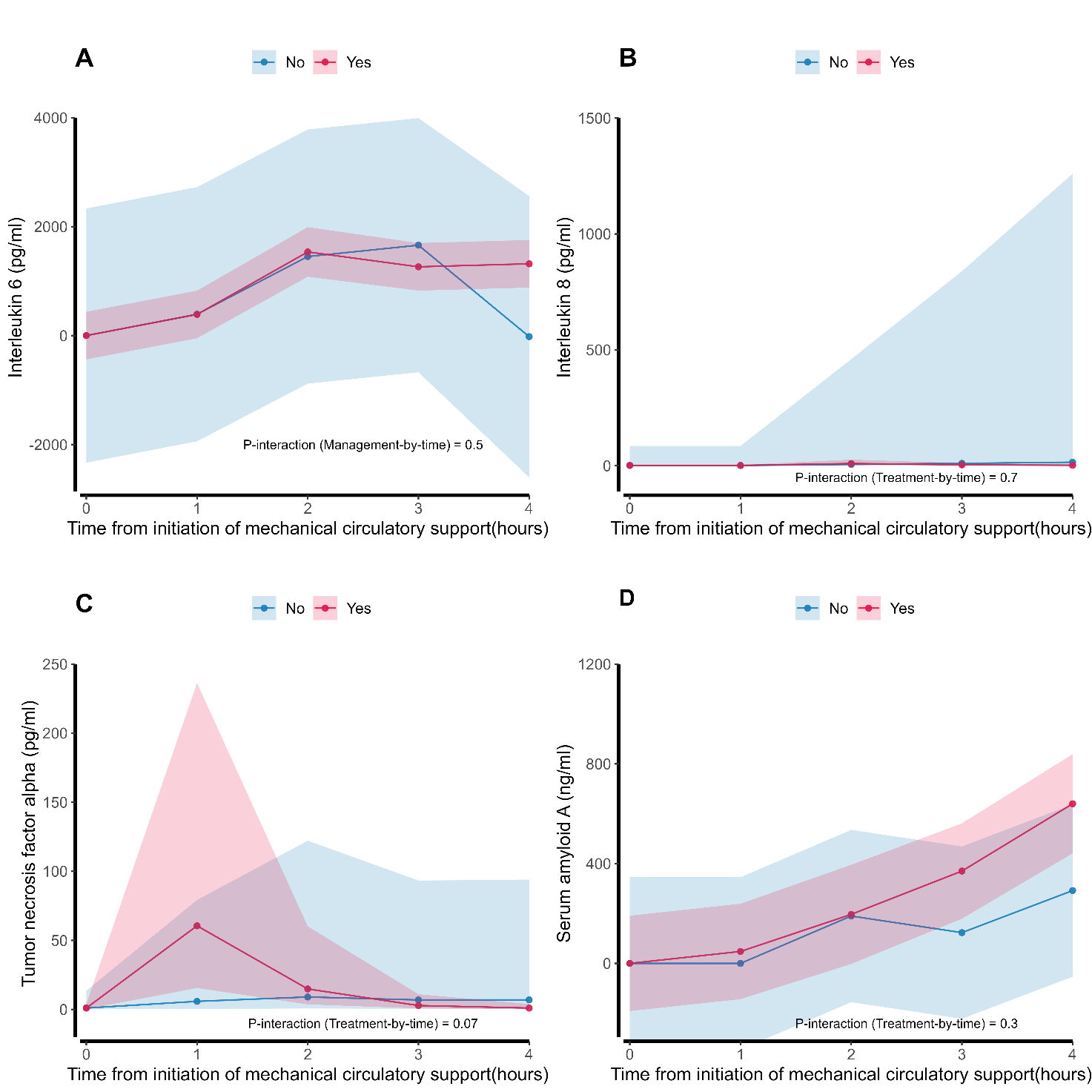

Supplement: Supplementary file 1 — Additional file 1. Supplementary Figures. [file 40635_2024_625_MOESM1_ESM.docx]
